# A legacy in print: The publication impact of Professor Rinaldo Bellomo

**DOI:** 10.1016/j.ccrj.2025.100124

**Published:** 2025-10-14

**Authors:** Michael Bailey, Ary Serpa Neto, Paul J. Young

**Affiliations:** a Australian and New Zealand Intensive Care Research Centre, School of Public Health and Preventive Medicine, Monash University, Melbourne, Victoria, Australia; b Department of Intensive Care, Austin Hospital, Melbourne, Australia; c Department of Critical Care, Melbourne Medical School, University of Melbourne, Austin Hospital, Melbourne, Australia; d Department of Critical Care Medicine, Hospital Israelita Albert Einstein, São Paulo, Brazil; e Intensive Care Unit, Wellington Hospital, Wellington, New Zealand; f Medical Research Institute of New Zealand, Wellington, New Zealand

**Keywords:** Rinaldo Bellomo, Publication metrics, Critical care research, Academic impact, Scientometric analysis

## Abstract

Professor Rinaldo Bellomo's legacy as a world-leading clinician-scientist is unmatched in the field of critical care. This tribute explores the depth, breadth, and global influence of his publication record, spanning more than four decades. With over 2000 peer-reviewed articles, 81 elite journal publications, and a remarkable H-index above 200, Rinaldo's academic contributions helped shape international definitions of acute kidney injury and sepsis, advanced critical care nephrology, and guided fluid resuscitation practice worldwide. Through editorial leadership, strategic authorship, and mentorship, he transformed the landscape of intensive care research.

## 1 Introduction

Several heartfelt reflections have already been published across the international critical care literature, capturing the breadth of Rinaldo's legacy as a clinician-scientist, mentor, and editor.[1], [2], [3], [4], [5] This document complements those tributes by systematically exploring the enduring impact of his published work, highlighting how his authorship, leadership, and editorial vision helped define the global trajectory of intensive care research.

With more than 2000 peer-reviewed scientific articles indexed in PubMed, the world's leading database for biomedical research, Rinaldo Bellomo's academic output reflects an extraordinary and sustained intellectual contribution. His publishing career began in 1981, while he was still an undergraduate at the University of Milan, with a paper in Clinical Toxicology that demonstrated how combining neurophysiological testing with biochemical markers could improve the early detection of occupational neurotoxicity.

**Fig. 1:**
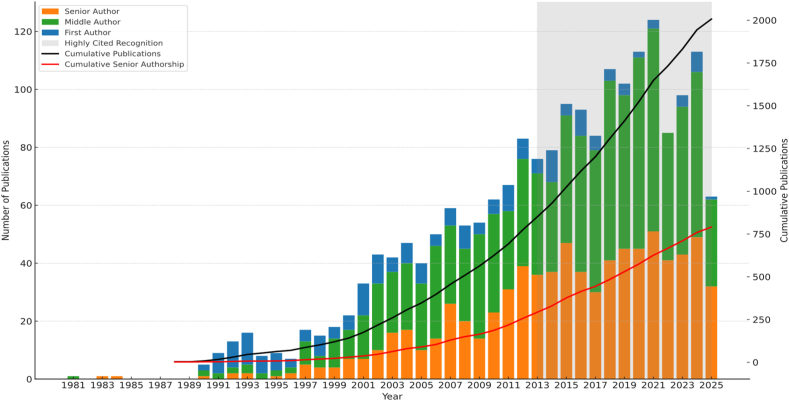
Rinaldo Bellomo's publication history displaying annual and cumulative counts according to authorship role.

Although he published intermittently in the 1980s, Rinaldo's academic career accelerated in the 1990s. By the turn of the millennium, he had coauthored over 120 manuscripts, with more than half of them as the principal author. The 2000s marked a period of transformation, as his annual publication rate rose steadily from 22 papers in 2000 to 54 by 2009, culminating in 443 papers across the decade. His impact reached new heights in the 2010s, when he was recognised globally as one of the most cited and productive researchers in medicine, further cementing his place as the most prolific researcher in the history of critical care. Between 2010 and 2019, he authored 848 papers, including 65 as first author and nearly 400 as senior author. His extraordinary output continued into the next decade. In 2021 alone, he published 124 papers, and between 2020 and 2025, he averaged over 100 publications each year. Across his career, Rinaldo first-authored more than 210 manuscripts and served as senior author on more than 750. Yet these numbers tell only part of the story. As his career advanced, his authorship shifted from the front line to the helm, moving from first-author to senior-author roles (Fig. 1). This evolution reflected his growing influence as a mentor, thought leader, and architect of global ICU research collaborations, shaping the careers of others as profoundly as he shaped the science itself.

Some have argued that publishing at this scale inevitably violates authorship criteria. Rinaldo respectfully disagreed, and let his record speak for itself. He balanced a clinical workload with rigorous scholarship, fulfilling all authorship responsibilities: conceptualisation, critical revision, final approval, and accountability. He considered research to be a craft rather than a duty, and approached publication as both science and strategy, understanding editorial expectations, mastering peer review diplomacy, and, above all, writing with precision and clarity.

## 2 Making an impact

**Fig. 2:**
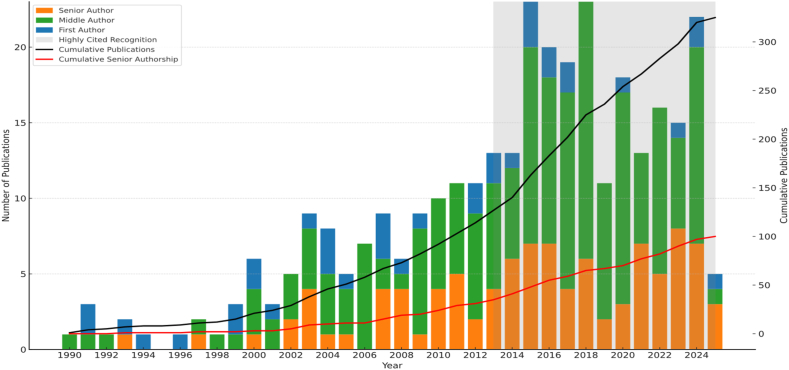
Rinaldo's publication history displaying annual and cumulative counts according to high-impact publications (2024 Journal Impact factor >10).

Rinaldo embodied Albert Einstein's maxim: “You have to learn the rules of the game. And then you have to play better than anyone else.” When it came to publications, Rinaldo played better than most. His legacy rests not only on the number of papers he authored, but on the influence they had across clinical practice and research. He had an exceptional instinct for recognising the potential of each manuscript and identifying the most effective strategy to realise that potential. Valuing the role of rigorous peer review, Rinaldo typically submitted strong manuscripts to journals higher up the academic hierarchy, confident that if they weren't accepted, thoughtful reviewer critique would enhance the final product. When targeting the highest-impact journals, he consistently sought presubmission feedback from foremost experts in the field, most of whom were trusted colleagues. Over the course of his career, he built an ever-growing portfolio of impactful publications, culminating in 325 papers in leading journals, including more than 100 as senior author. Notably, over half of these were published in the final decade of his life (Fig. 2).

**Fig. 3:**
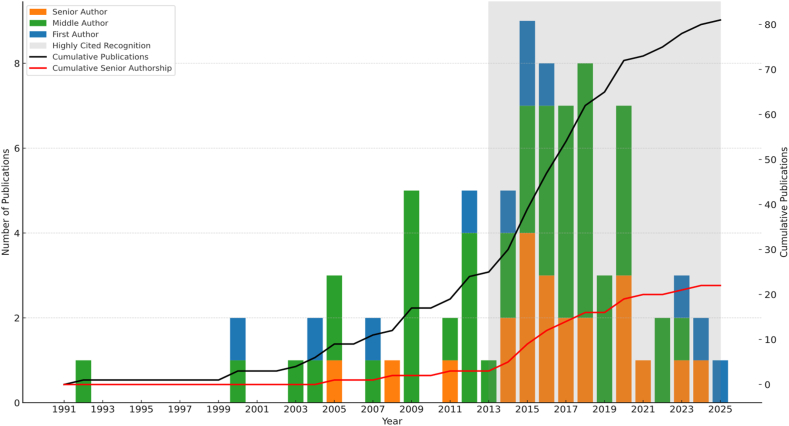
Rinaldo's publication history displaying annual and cumulative counts according to authorship role for elite publications (NEJM, JAMA or The Lancet).

The *New England Journal of Medicine*, *JAMA*, and *The Lancet* are widely recognised as the most prestigious medical journals, esteemed for their rigorous peer review, high impact factors, international readership, and consistent publication of studies that shape clinical practice and influence global health policy. Like many aspiring researchers, Rinaldo began his career hoping for just a single publication in a journal of this calibre. His first elite publication appeared in *The Lancet* in 1992. Although eight years passed before his next, he soon became a regular contributor. Between 2015 and 2020, he amassed an extraordinary 42 elite publications, at a rate of around 7 per year. Over the course of his career, he achieved a total of 81 publications in these leading journals, including 32 as either first or senior author, firmly placing him among the most elite medical researchers in the history of academic medicine (Fig. 3).

## 3 Journals

Rinaldo published in more than 280 medical journals, with over half of his articles appearing in critical care-focused outlets (eFig. 1). No journal featured his work more prominently than *Critical Care and Resuscitation (CCR)* (eFig. 2), where his contributions increased significantly after becoming Editor-in-Chief in 2007. In this role, Rinaldo transformed the journal into a cornerstone of Australasian critical care research. He strengthened regional research visibility by championing quality studies from Australia, New Zealand, and beyond, offering a platform for important work that may otherwise have struggled to reach a global audience. His editorial approach emphasised both academic rigour and practical relevance, ensuring that the journal published research that was methodologically sound and clinically meaningful. Rinaldo was a generous and insightful mentor, guiding early-career authors with constructive feedback that shaped not only their manuscripts but also their development as investigators. Under his leadership, *CCR* expanded its scope to include clinical trials, education, systems-based research, and more, reflecting his vision of a journal that both documented and advanced the practice of intensive care medicine.

## 4 Authors

Over the course of his career, Rinaldo published with more than 5000 individual coauthors, spanning 74 countries and representing over 3800 distinct institutional affiliations. A summary of his most frequent collaborators is presented in eFigure3. Rinaldo was deeply collaborative and deliberately inclusive when it came to co-authorship. He understood that being part of a high-impact publication could play a pivotal role in shaping the academic careers of younger researchers, and was intentional in offering such opportunities. His generosity in sharing authorship reflected not only his commitment to advancing science, but also his desire to foster the next generation of clinical researchers.

## 5 Landmark publications

Rinaldo Bellomo's publication metrics speak volumes about both the scale and influence of his academic work. The H-index, which identifies the number of papers (H) with at least H citations, is a widely used measure of scientific impact. With an H-index above 200, Rinaldo is part of an exceptionally rare group of researchers whose work has shaped clinical thinking across multiple domains. His work laid the foundation for international definitions, clinical guidelines, and evidence-based practice across intensive care and nephrology (eTable 1). His i10-index of 1423, including more than 500 first-authored or senior-authored papers (eTable 2), further illustrates a career of remarkable reach, depth, and sustained scholarly engagement.

Throughout his career, Rinaldo produced a series of landmark publications that transformed global understanding of acute kidney injury (AKI), fluid resuscitation, sepsis, and the relationship between cardiac and renal dysfunction. Rinaldo's early first-author papers in *Intensive Care Medicine* laid the groundwork for his later leadership in the development of renal support therapies. A pivotal 2001 consensus paper titled *Acute renal failure: time for consensus* initiated efforts to harmonise AKI terminology, helping to establish the intellectual basis for global frameworks such as Risk, Injury, Failure, Loss, End-stage kidney disease (RIFLE) and Kidney Disease: Improving Global Outcomes (KDIGO).^6^

In 2004, he was first author of a key consensus paper from the Acute Dialysis Quality Initiative, published in *Critical Care*. This publication introduced the RIFLE criteria, which became the first internationally accepted definition of AKI and transformed how the syndrome was staged and monitored in both research and clinical settings.^7^

That same year, Rinaldo served as senior author on the saline versus albumin fluid evaluation (SAFE) study, an international multicentre trial published in *The New England Journal of Medicine*, comparing albumin to saline for fluid resuscitation. The trial demonstrated no difference in mortality outcomes between the two fluids, helping reshape fluid therapy practices in critical care.^8^

In 2005, he led a major multinational study of AKI published in *JAMA*, which quantified the incidence and outcomes of AKI in critically ill patients and demonstrated its key role in determining prognosis.^9^

Alongside his contributions to kidney and fluid therapies, Rinaldo played a pivotal role in developing and evaluating the Medical Emergency Team (MET) model to improve the recognition of, and response to, patient deterioration in hospital. At Austin Health, he helped implement one of Australia's first ICU-led MET systems and led a series of influential studies demonstrating reductions in cardiac arrests, unplanned ICU admissions, and in-hospital mortality. To rigorously evaluate the model, he co-led the Medical Emergency Response and Intervention Trial (MERIT), a 23-hospital cluster trial published in *The Lancet* in 2005,^10^ marking Australasia's first ICU-led cluster randomised controlled trial and a key step in the global adoption of rapid response systems.

In 2008, Rinaldo coauthored a seminal paper in the *Journal of the American College of Cardiology* that proposed a structured classification of the cardiorenal syndrome. This framework provided a practical and enduring model to describe the complex interactions between heart and kidney dysfunction.^11^

His contributions to fluid management and kidney injury continued with a 2010 review in *Nature Reviews Nephrology* on fluid balance in AKI,^12^ and a 2017 consensus paper introducing the concept of acute kidney disease.^13^ The latter work, also developed through the Acute Dialysis Quality Initiative, expanded understanding of kidney injury as a clinical spectrum that extends beyond the acute phase.

The 2012 *Lancet* Seminar on AKI, coauthored with Claudio Ronco and John Kellum, remains one of the most influential summaries in the field.^14^ It synthesised decades of research into a clinically actionable framework that continues to guide both clinical care and research.

That same year, Rinaldo coauthored the Crystalloid versus Hydroxyethyl Starch Trial (CHEST), published in *The New England Journal of Medicine*, which demonstrated that hydroxyethyl starch was associated with increased harm compared to saline. This study contributed to a global shift away from synthetic colloids in ICU resuscitation.^15^

By 2014 and 2015, Rinaldo had turned his attention to sepsis epidemiology and diagnostic definitions. As senior author on two high-impact papers, he showed that sepsis-related mortality was decreasing across Australia and New Zealand (*JAMA*, 2014),^16^ and highlighted the limitations of the systemic inflammatory response syndrome (SIRS) criteria in detecting severe infections (*NEJM*, 2015).^17^ These studies directly contributed to the development of the Sepsis-3 definitions, published in 2016. Rinaldo was a key contributor to that international effort and is a named coauthor on the 2016 *JAMA* paper that introduced the new sepsis and septic shock definitions.^18^ This paper has since become one of the most cited publications in medical literature, with more than 30,000 citations.

In 2015, Rinaldo played a central role in the Acute Kidney Injury–Epidemiologic Prospective Investigation (AKI-EPI), a global multicentre observational study published in *Intensive Care Medicine*.^19^ This work updated the global epidemiology of AKI, revealing significant variation in incidence and outcomes between regions, and further emphasised the need for standardised definitions.

His 2019 *Lancet* review on AKI, which was once again coauthored with two of his most enduring collaborators, Ronco and Kellum, provided a comprehensive update on the condition's pathophysiology, biomarkers, and evolving therapies.^20^ It remains a cornerstone reference in nephrology and critical care.

Even in his final years, Rinaldo continued to challenge prevailing clinical assumptions. In 2020, he coauthored the Vitamin C, Hydrocortisone and Thiamine in patients with Septic Shock (VITAMINS) trial published in *JAMA*, which showed that the addition of vitamin C and thiamine to hydrocortisone did not improve outcomes in septic shock.^21^ The study redefined the evidence base for adjunctive therapies in sepsis and reinforced the importance of rigorous testing for widely adopted interventions.

Professor Rinaldo Bellomo's publishing legacy is remarkable not only for its volume but for its lasting impact on clinical practice and academic medicine. He set a new benchmark for critical care research in Australia and internationally, mentoring a generation of clinician-scientists and helping to define excellence in intensive care scholarship. His scientific contributions fundamentally changed the way we understand and manage critical illness. Yet it was his unwavering commitment to collaboration, mentorship, and patient-focused research that truly defined his legacy. He empowered countless researchers, championed work that improved lives, and elevated the global standing of Australian and New Zealand critical care. His passing marks an immeasurable loss to the field, but his influence will continue to shape medicine for years to come.^1^

## CRediT authorship contribution statement

MB: Conceptualization; Methodology; Formal analysis; Writing original draft. ASN: Methodology; Validation; Writing review and editing. PY: Methodology; Validation; Writing review and editing.

## Declaration of competing interest

The authors declare the following financial interests/personal relationships which may be considered as potential competing interests: Paul Young & Ary Serpa Neto are currently editors in chief for Critical Care and Resuscitation. Given their role as editors, they had no involvement in the peer review of this article and had no access to information regarding its peer review. Full responsibility for the editorial process for this article was delegated to another journal editor. If there are other authors, they declare that they have no known competing financial interests or personal relationships that could have appeared to influence the work reported in this paper.

## Footnotes

1 Publication metrics for this tribute were determined as of 1 July 2025. Remarkably, even posthumously, Professor Bellomo continued to publish at an extraordinary rate, with over 35 publications appearing since his passing.
