# Supplementary material for: A legacy in print: The publication impact of Professor Rinaldo Bellomo
Source: Crit Care Resusc. 2025 Oct 14;27(3):100124. doi: 10.1016/j.ccrj.2025.100124 (PMC12847636; doi:10.1016/j.ccrj.2025.100124)
Supplement: Multimedia component 1 [file mmc1.docx]

**Supplementary Appendix**

**Title:** **A Legacy in Print: The Publication Impact of Professor Rinaldo Bellomo**

**Table of Figures**

[**eFigure 1. Fields of medicine that Rinaldo Bellomo published in** 2](#_Toc204153248)

[**eFigure 2 Top 20 Journals (2024 Impact Factor) for Rinaldo Bellomo's publications** 2](#_Toc204153249)

[**eFigure 3 Top 50 most prolific co-authors for Rinaldo Bellomo** 3](#_Toc204153250)

**Table of Tables**

[**eTable 1 Top 15 most heavily cited publications for Rinaldo Bellomo** 4](#_Toc204144170)

[**eTable 2 Rinaldo Bellomo’s Top 500 cited publications as either first or senior author** 5](#_Toc204144171)

**eFigure 1. Fields of medicine that Rinaldo Bellomo published in**


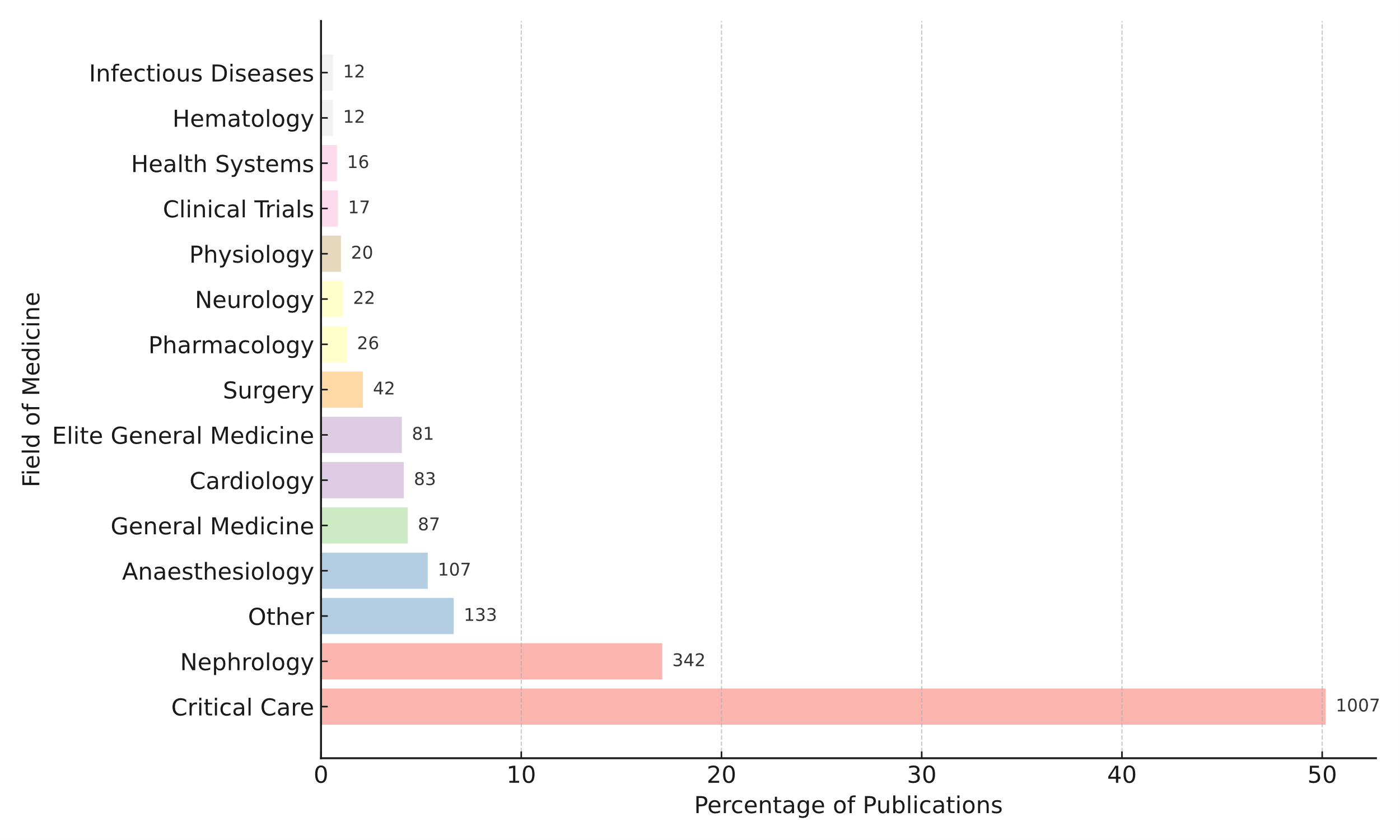


**eFigure 2. Top 20 Journals (2024 Impact Factor) for Rinaldo Bellomo's publications**


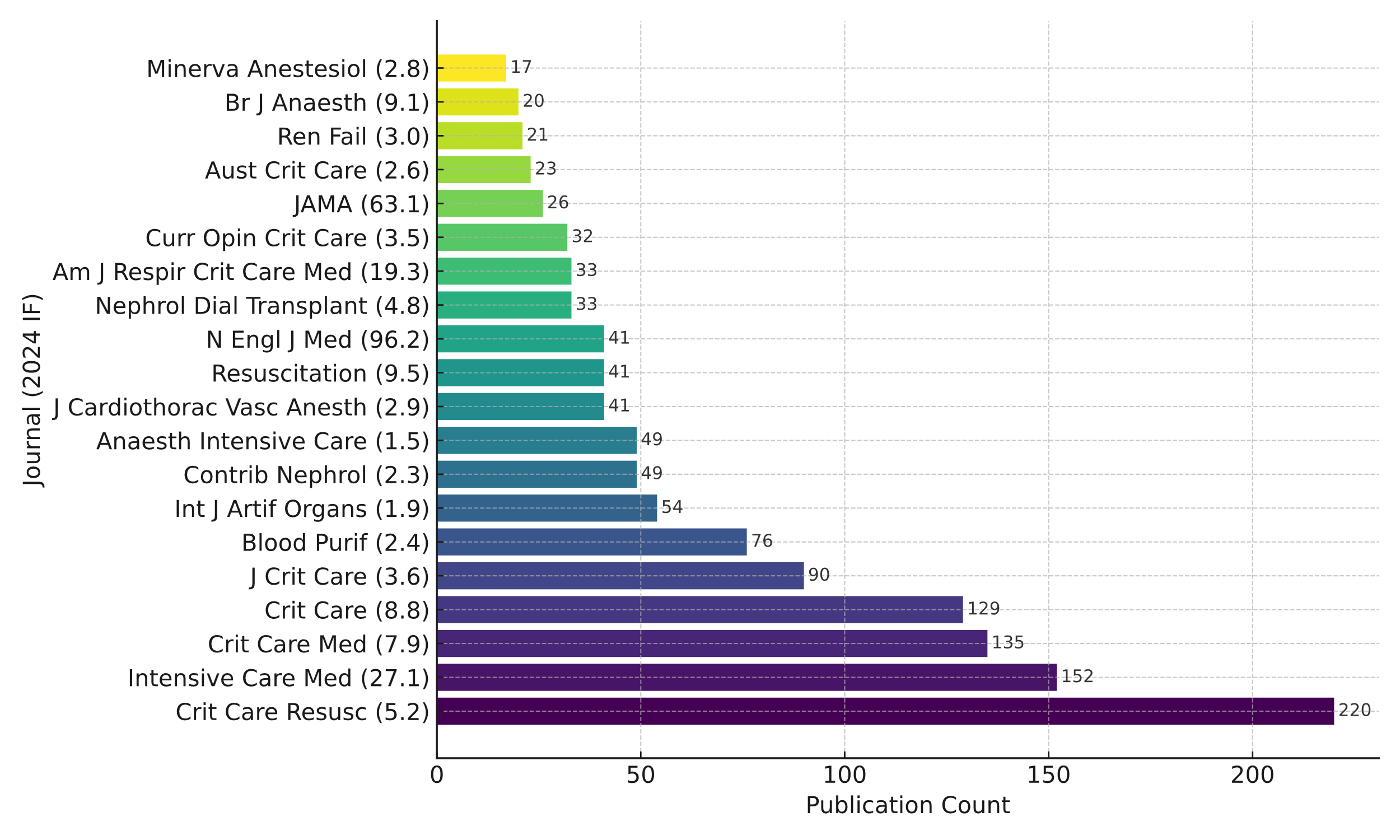


**eFigure 3. Top 50 most prolific co-authors for Rinaldo Bellomo**

**eTable 1. Top 15 most heavily cited publications for Rinaldo Bellomo**

| Year | Shortened Title | Journal | Citations | Citations  Per Year |
| --- | --- | --- | --- | --- |
| 2016 | The third international consensus definitions for sepsis (Sepsis-3) | JAMA | 29923 | 2992 |
| 2004 | AKI definition, outcome measures, ADQI consensus | Critical Care | 8773 | 399 |
| 2005 | Global AKI incidence in ICU patients | JAMA | 5547 | 264 |
| 2004 | SAFE study: albumin vs saline for fluid resuscitation | NEJM | 3793 | 172 |
| 2008 | Classification of cardiorenal syndrome | J Am Coll Cardiol | 3069 | 171 |
| 2015 | AKI-EPI study: global epidemiology of AKI | ICM | 3038 | 276 |
| 2000 | Haemofiltration dosing in acute renal failure | Lancet | 2371 | 91 |
| 2014 | Goal-directed resuscitation in septic shock | NEJM | 2222 | 185 |
| 2012 | Acute kidney injury (seminar) | Lancet | 2211 | 158 |
| 2012 | CHEST trial: hydroxyethyl starch vs saline | NEJM | 2166 | 155 |
| 2014 | Declining mortality in sepsis (ANZ cohort) | JAMA | 2149 | 179 |
| 2019 | Acute kidney injury (state-of-the-art review) | Lancet | 1814 | 259 |
| 2009 | CRRT intensity in critically ill patients | NEJM | 1755 | 103 |
| 2009 | NGAL in AKI: systematic review and meta-analysis | Am J Kidney Dis | 1732 | 102 |
| 2015 | SIRS criteria limitations in defining sepsis | NEJM | 1664 | 151 |

*** Google Scholar citation at July 1 2025**

**^ IF = Journal Impact factor in 2024**

**eTable 2. Rinaldo Bellomo’s Top 500 cited publications as either first or senior author**

| Order | Short Title | Journal | Year | Role | Citations* | IF^ |
| --- | --- | --- | --- | --- | --- | --- |
| 1 | Cardiorenal syndrome | J Am Coll Cardiol | 2008 | Senior | 3069 | 21.7 |
| 2 | Acute kidney injury | Lancet | 2012 | Primary | 2211 | 79.3 |
| 3 | Mortality related to severe sepsis and septic | JAMA | 2014 | Senior | 2149 | 63.1 |
| 4 | Acute kidney injury | Lancet | 2012 | Primary | 1814 | 79.3 |
| 5 | Systemic inflammatory response syndrome criteria | N Engl J Med | 2015 | Senior | 1664 | 96.2 |
| 6 | Early acute kidney injury and sepsis: a | Crit Care | 2008 | Senior | 972 | 8.8 |
| 7 | Rapid-response teams | N Engl J Med | 2011 | Senior | 930 | 96.2 |
| 8 | A comparison of the RIFLE and AKIN criteria for | Nephrol Dial Transplant | 2008 | Senior | 874 | 4.8 |
| 9 | Stress hyperglycemia: an essential survival | Crit Care Med | 2013 | Senior | 871 | 7.7 |
| 10 | Angiotensin II for the Treatment of Vasodilatory | N Engl J Med | 2017 | Senior | 869 | 96.2 |
| 11 | Acute kidney injury in sepsis | Intensive Care Med | 2017 | Primary | 822 | 27.1 |
| 12 | A multi-centre evaluation of the RIFLE criteria | Nephrol Dial Transplant | 2008 | Senior | 771 | 4.8 |
| 13 | Cardiac surgery-associated acute kidney injury: | Nat Rev Nephrol | 2017 | Senior | 704 | 23.6 |
| 14 | Prospective controlled trial of effect of medical | Crit Care Med | 2004 | Primary | 688 | 7.7 |
| 15 | Fluid balance and acute kidney injury | Nat Rev Nephrol | 2010 | Senior | 620 | 23.6 |
| 16 | Interpreting the mechanisms of continuous renal | Artif Organs | 2003 | Senior | 613 | 2.3 |
| 17 | Very old patients admitted to intensive care in | Crit Care | 2009 | Senior | 588 | 8.8 |
| 18 | Sepsis-associated hyperlactatemia | Crit Care | 2014 | Senior | 565 | 8.8 |
| 19 | Defining acute renal failure: physiological | Intensive Care Med | 2004 | Primary | 559 | 27.1 |
| 20 | Continuous veno-venous hemofiltration with | Crit Care Med | 1993 | Primary | 541 | 7.7 |
| 21 | Continuous versus intermittent renal replacement | Crit Care Med | 2008 | Senior | 502 | 7.7 |
| 22 | Renal blood flow in experimental septic acute | Kidney Int | 2006 | Senior | 488 | 8.5 |
| 23 | Pathophysiology of septic acute kidney injury: | Crit Care Med | 2008 | Senior | 485 | 7.7 |
| 24 | Defining and classifying acute renal failure: | Intensive Care Med | 2007 | Primary | 483 | 27.1 |
| 25 | Plasma and urine neutrophil gelatinase-associated | Intensive Care Med | 2010 | Senior | 479 | 27.1 |
| 26 | Long-term risk of adverse outcomes after acute | Kidney Int | 2019 | Senior | 476 | 8.5 |
| 27 | Fluid management for the prevention and | Nat Rev Nephrol | 2014 | Senior | 406 | 23.6 |
| 28 | Why we should be wary of single-center trials | Crit Care Med | 2009 | Primary | 383 | 7.7 |
| 29 | The pathophysiology of cardiac surgery-associated | Int J Artif Organs | 2008 | Primary | 383 | 1.4 |
| 30 | Dexmedetomidine vs. haloperidol in delirious, | Crit Care | 2009 | Senior | 382 | 8.8 |
| 31 | Arterial hyperoxia and in-hospital mortality | Crit Care | 2011 | Primary | 381 | 8.8 |
| 32 | Changes in the incidence and outcome for early | Crit Care | 2007 | Senior | 376 | 8.8 |
| 33 | Myocardial cell injury in septic shock | Crit Care Med | 1999 | Senior | 371 | 7.7 |
| 34 | A rational approach to fluid therapy in sepsis | Br J Anaesth | 2016 | Senior | 368 | 6.9 |
| 35 | The histopathology of septic acute kidney injury: | Crit Care | 2008 | Senior | 364 | 8.8 |
| 36 | Acute renal failure: time for consensus | Intensive Care Med | 2001 | Primary | 323 | 27.1 |
| 37 | Bench-to-bedside review: lactate and the kidney | Crit Care | 2002 | Primary | 314 | 8.8 |
| 38 | Clinical review: early patient mobilization in | Crit Care | 2013 | Senior | 311 | 8.8 |
| 39 | The rise and fall of NGAL in acute kidney injury | Blood Purif | 2014 | Senior | 311 | 2.2 |
| 40 | Oliguria as predictive biomarker of acute kidney | Crit Care | 2011 | Senior | 304 | 8.8 |
| 41 | Development and implementation of a high-quality | J Crit Care | 2006 | Senior | 286 | 2.9 |
| 42 | Intravenous fluid therapy in critically ill adults | Nat Rev Nephrol | 2018 | Senior | 273 | 23.6 |
| 43 | Acquired bloodstream infection in the intensive | Crit Care | 2011 | Senior | 262 | 8.8 |
| 44 | Loop diuretics in the management of acute renal | Crit Care Resusc | 2007 | Senior | 258 | 1.4 |
| 45 | Outcomes in Patients with Vasodilatory Shock and | Crit Care Med | 2018 | Senior | 253 | 7.7 |
| 46 | Dynamic lactate indices as predictors of outcome | Crit Care | 2011 | Senior | 253 | 8.8 |
| 47 | Vital organ blood flow during hyperdynamic sepsis | Chest | 2003 | Senior | 249 | 10.3 |
| 48 | Variability of antibiotic concentrations in | Crit Care Med | 2012 | Senior | 241 | 7.7 |
| 49 | Effect of an automated notification system for | Crit Care | 2017 | Senior | 239 | 8.8 |
| 50 | Stress hyperlactataemia: present understanding | Lancet Diabetes Endocrinol | 2014 | Senior | 237 | 28 |
| 51 | Timing of onset and burden of persistent critical | Lancet Respir Med | 2016 | Senior | 235 | 39.6 |
| 52 | Unmeasured anions in critically ill patients: can | Crit Care Med | 2003 | Senior | 230 | 7.7 |
| 53 | Acute renal failure and multiple organ | Int J Artif Organs | 2002 | Senior | 229 | 1.4 |
| 54 | Continuous veno-venous hemofiltration without | Intensive Care Med | 2000 | Senior | 225 | 27.1 |
| 55 | Early blood glucose control and mortality in | Crit Care Med | 2009 | Senior | 215 | 7.7 |
| 56 | Urinary biochemistry and microscopy in septic | Am J Kidney Dis | 2006 | Senior | 214 | 6.8 |
| 57 | Vasoactive drugs and acute kidney injury | Crit Care Med | 2008 | Primary | 213 | 7.7 |
| 58 | Plasma-Lyte 148 vs 0.9% saline for fluid | J Crit Care | 2012 | Senior | 211 | 2.9 |
| 59 | Preliminary experience with high-volume | Kidney Int Suppl | 1998 | Primary | 208 | 2 |
| 60 | A multi-center evaluation of early acute kidney | Ren Fail | 2008 | Senior | 208 | 2.9 |
| 61 | Trace element and vitamin concentrations and | Crit Care Med | 1999 | Senior | 204 | 7.7 |
| 62 | Effects of norepinephrine on the renal | Am J Respir Crit Care Med | 1999 | Primary | 203 | 19.3 |
| 63 | The effect of intensive plasma water exchange by | Am J Respir Crit Care Med | 2000 | Primary | 201 | 19.3 |
| 64 | Continuous renal replacement therapy: recent | Nat Rev Nephrol | 2010 | Senior | 200 | 23.6 |
| 65 | Acute kidney injury in the ICU: from injury to | Ann Intensive Care | 2017 | Primary | 199 | 5.8 |
| 66 | A Randomized Controlled Trial of Regional Citrate | Crit Care Med | 2015 | Senior | 198 | 7.7 |
| 67 | Targeted therapeutic mild hypercapnia after | Resuscitation | 2016 | Senior | 195 | 6.5 |
| 68 | Continuous renal replacement therapy in the | Intensive Care Med | 1999 | Primary | 194 | 27.1 |
| 69 | Validation of the Kidney Disease Improving Global | Clin J Am Soc Nephrol | 2014 | Senior | 193 | 7.2 |
| 70 | A controlled trial of electronic automated | Crit Care Med | 2012 | Primary | 193 | 7.7 |
| 71 | Membranous nephropathy and thromboembolism: is | Nephron | 1993 | Primary | 191 | 2.3 |
| 72 | Noradrenaline and the kidney: friends or foes? | Crit Care | 2001 | Primary | 191 | 8.8 |
| 73 | Therapeutic hypothermia: benefits, mechanisms and | Injury | 2011 | Senior | 187 | 3.3 |
| Order | **Short Title** | **Journal** | **Year** | **Role** | **Citations*** | **IF^** |
| 74 | Nurse’s attitudes to a medical emergency | BMJ Quality & Safety | 2006 | Senior | 187 | 6.5 |
| 75 | Measurement of renal blood flow by phase-contrast | Crit Care Med | 2012 | Senior | 182 | 7.7 |
| 76 | Cystatin C in acute kidney injury | Curr Opin Crit Care | 2010 | Senior | 181 | 3.5 |
| 77 | Renal blood flow and function during recovery | Intensive Care Med | 2007 | Senior | 180 | 27.1 |
| 78 | Extracorporeal blood treatment (EBT) methods in | Int J Artif Organs | 2005 | Primary | 179 | 1.4 |
| 79 | Sepsis-associated acute kidney injury: | Semin Nephrol | 2015 | Senior | 178 | 4.2 |
| 80 | Myoglobin clearance by super high-flux | Crit Care | 2005 | Senior | 176 | 8.8 |
| 81 | Age of red blood cells and transfusion in | Ann Intensive Care | 2013 | Senior | 176 | 5.8 |
| 82 | Renin and Survival in Patients Given Angiotensin | Am J Respir Crit Care Med | 2020 | Primary | 176 | 19.3 |
| 83 | Urinary biomarkers in septic acute kidney injury | Intensive Care Med | 2007 | Senior | 174 | 27.1 |
| 84 | The epidemiology of acute renal failure: 1975 | Curr Opin Crit Care | 2006 | Primary | 172 | 3.5 |
| 85 | Erythropoietin (EPO) in acute kidney injury | Ann Intensive Care | 2011 | Senior | 171 | 5.8 |
| 86 | The cardiorenal syndrome | Blood Purif | 2009 | Senior | 168 | 2.2 |
| 87 | Phase II, randomized, controlled trial of | Crit Care Med | 2007 | Senior | 162 | 7.7 |
| 88 | Extended Daily Dialysis Versus Continuous Renal | Am J Kidney Dis | 2015 | Senior | 161 | 6.8 |
| 89 | High protein intake during continuous | Int J Artif Organs | 2002 | Primary | 161 | 1.4 |
| 90 | Renal histopathology during experimental septic | Crit Care Med | 2014 | Senior | 158 | 7.7 |
| 91 | Current oxygen management in mechanically | J Crit Care | 2013 | Senior | 153 | 2.9 |
| 92 | Intrarenal blood flow distribution in | Crit Care Med | 2003 | Senior | 152 | 7.7 |
| 93 | Continuous haemofiltration in the intensive care | Crit Care | 2000 | Primary | 151 | 8.8 |
| 94 | Postoperative serious adverse events in a | Med J Aust | 2002 | Primary | 151 | 5 |
| 95 | Hemoperfusion: technical aspects and state of the | Crit Care | 2022 | Senior | 150 | 8.8 |
| 96 | Anticoagulant regimens in acute continuous | Intensive Care Med | 1993 | Primary | 150 | 27.1 |
| 97 | Pre-dilution vs. post-dilution during continuous | Nephron Clin Pract | 2003 | Senior | 149 | 1.471 |
| 98 | Structure and Function of the Kidney in Septic | Am J Respir Crit Care Med | 2016 | Senior | 146 | 19.3 |
| 99 | Characteristics and outcomes of patients | Crit Care Med | 2008 | Senior | 146 | 7.7 |
| 100 | Incidence, risk factors and outcome associations | Anaesth Intensive Care | 2012 | Senior | 145 | 1.1 |
| 101 | Ionized calcium concentration and outcome in | Crit Care Med | 2011 | Senior | 144 | 7.7 |
| 102 | Early diagnosis of acute kidney injury | Curr Opin Crit Care | 2007 | Senior | 142 | 3.5 |
| 103 | A multicenter study on the effect of continuous | Crit Care | 2015 | Senior | 140 | 8.8 |
| 104 | Acute kidney injury after cardiac arrest | Resuscitation | 2012 | Senior | 140 | 6.5 |
| 105 | A systematic review of urinary findings in | Crit Care Med | 2007 | Senior | 139 | 7.7 |
| 106 | The cytokine network in the critically ill | Anaesth Intensive Care | 1992 | Primary | 139 | 1.1 |
| 107 | Documentation of clinical review and vital signs | Med J Aust | 2008 | Senior | 137 | 5 |
| 108 | Coupled plasma filtration adsorption | Blood Purif | 2002 | Primary | 136 | 2.2 |
| 109 | Coupled plasma filtration adsorption | Intensive Care Med | 2003 | Primary | 136 | 27.1 |
| 110 | The medical emergency team and end-of-life care: | Crit Care Resusc | 2007 | Senior | 136 | 1.4 |
| 111 | Norepinephrine and vital organ blood flow during | Intensive Care Med | 2003 | Senior | 134 | 27.1 |
| 112 | Continuous versus intermittent renal replacement | Kidney Int Suppl | 1998 | Primary | 134 | 2 |
| 113 | Septic acute kidney injury: new concepts | Nephron Exp Nephrol | 2008 | Primary | 132 | 2.3 |
| 114 | Near infrared spectroscopy (NIRS) of the thenar | Ann Intensive Care | 2012 | Senior | 131 | 5.8 |
| 115 | A prospective evaluation of urine microscopy in | Nephrol Dial Transplant | 2012 | Senior | 130 | 4.8 |
| 116 | Conservative oxygen therapy in mechanically | Crit Care Med | 2014 | Senior | 130 | 7.7 |
| 117 | Transvisceral lactate fluxes during early | Chest | 1996 | Primary | 129 | 10.3 |
| 118 | Out of hospital outcome and quality of life in | Intensive Care Med | 1997 | Senior | 128 | 27.1 |
| 119 | Defining, quantifying, and classifying acute | Crit Care Clin | 2005 | Primary | 127 | 2.8 |
| 120 | Vitamin B1 in critically ill patients: needs and | Clin Chem Lab Med | 2017 | Senior | 127 | 3.2 |
| 121 | Cardiopulmonary bypass-associated acute kidney | Contrib Nephrol | 2007 | Senior | 124 | 2.3 |
| 122 | Severe acute renal failure: a comparison of acute | Nephron | 1995 | Primary | 124 | 2.3 |
| 123 | Acute renal failure in the intensive care unit: | Nephrol Dial Transplant | 1996 | Primary | 123 | 4.8 |
| 124 | Glycemic control in the intensive care unit: why | Mayo Clin Proc | 2005 | Primary | 121 | 11 |
| 125 | Continuous venovenous hemofiltration without | ASAIO J | 2004 | Senior | 120 | 2.4 |
| 126 | A critique of fluid bolus resuscitation in severe | Crit Care | 2012 | Senior | 119 | 8.8 |
| 127 | Long-term effect of a Medical Emergency Team on | Resuscitation | 2007 | Senior | 119 | 6.5 |
| 128 | Continuous arteriovenous haemodiafiltration in | Intensive Care Med | 1991 | Primary | 118 | 27.1 |
| 129 | Sepsis-Induced Acute Kidney Injury | Crit Care Clin | 2015 | Senior | 118 | 2.8 |
| 130 | Cardiovascular monitoring tools: use and misuse | Curr Opin Crit Care | 2003 | Primary | 117 | 3.5 |
| 131 | Urinary biochemistry in experimental septic acute | Nephrol Dial Transplant | 2006 | Senior | 116 | 4.8 |
| 132 | Pre-morbid glycemic control modifies the | Intensive Care Med | 2016 | Senior | 115 | 27.1 |
| 133 | Circadian pattern of activation of the medical | Crit Care | 2005 | Senior | 115 | 8.8 |
| 134 | Coupled plasma filtration adsorption: rationale, | Blood Purif | 2003 | Senior | 113 | 2.2 |
| 135 | Coupled plasma filtration adsorption: rationale, | Contrib Nephrol | 2004 | Senior | 113 | 2.3 |
| 136 | The impact of premorbid diabetic status on the | Curr Opin Clin Nutr Metab Care | 2012 | Senior | 111 | 3 |
| 137 | Glycemic control in the ICU | Chest | 2011 | Senior | 109 | 10.3 |
| 138 | A prospective study of factors influencing the | Intensive Care Med | 2008 | Senior | 109 | 27.1 |
| 139 | Characteristics and outcomes of patients | J Crit Care | 2008 | Senior | 109 | 2.9 |
| 140 | Angiotensin I and angiotensin II concentrations | Crit Care | 2020 | Primary | 106 | 8.8 |
| 141 | Intensive care unit management of the critically | Cardiology | 2001 | Primary | 106 | 2.5 |
| 142 | Plasma-Lyte 148: A clinical review | World J Crit Care Med | 2016 | Senior | 106 | 1.1 |
| 143 | Contrast-enhanced ultrasound to evaluate changes | Crit Care | 2013 | Senior | 105 | 8.8 |
| 144 | Blood purification in the intensive care unit: | World J Surg | 2001 | Primary | 104 | 2.7 |
| 145 | Interleukin-6 and interleukin-8 extraction during | Ren Fail | 1995 | Primary | 104 | 2.9 |
| 146 | Mean arterial pressure and mean perfusion | J Crit Care | 2015 | Senior | 102 | 2.9 |
| 147 | Features and outcome of patients receiving | Resuscitation | 2010 | Senior | 102 | 6.5 |
| 148 | Histopathology of Septic Acute Kidney Injury: A | Crit Care Med | 2016 | Senior | 101 | 7.7 |
| Order | **Short Title** | **Journal** | **Year** | **Role** | **Citations*** | **IF^** |
| 149 | A prospective comparative study of continuous | Am J Kidney Dis | 1993 | Primary | 101 | 6.8 |
| 150 | Norepinephrine and vital organ blood flow | Intensive Care Med | 2002 | Senior | 99 | 27.1 |
| 151 | Urine biochemistry in septic and non-septic acute | J Crit Care | 2013 | Senior | 99 | 2.9 |
| 152 | A sheep model for the study of focal epilepsy | Epilepsia | 2002 | Senior | 99 | 6.6 |
| 153 | Renal blood flow, fractional excretion of sodium | Curr Opin Crit Care | 2012 | Senior | 98 | 3.5 |
| 154 | Mild Hypercapnia or Normocapnia after | N Engl J Med | 2023 | Senior | 97 | 96.2 |
| 155 | Magnesium status and magnesium therapy in cardiac | J Crit Care | 2017 | Senior | 97 | 2.9 |
| 156 | Near-Infrared Spectroscopy in Adult Cardiac | J Cardiothorac Vasc Anesth | 2017 | Senior | 95 | 2.3 |
| 157 | Combination of biomarkers for diagnosis of acute | Ren Fail | 2015 | Senior | 95 | 2.9 |
| 158 | Estimation of fluid status changes in critically | J Crit Care | 2012 | Senior | 95 | 2.9 |
| 159 | A pilot randomized controlled crossover study | Int J Artif Organs | 2007 | Senior | 94 | 1.4 |
| 160 | Diuretics in the management of acute kidney | Contrib Nephrol | 2007 | Senior | 94 | 2.3 |
| 161 | Postoperative blood pressure deficit and acute | Crit Care | 2016 | Senior | 93 | 8.8 |
| 162 | The need to reform our assessment of evidence | Philos Ethics Humanit Med | 2008 | Senior | 92 | 1.9 |
| 163 | The pursuit of a high central venous oxygen | Crit Care | 2008 | Primary | 92 | 8.8 |
| 164 | Mortality in Multicenter Critical Care Trials: An | Crit Care Med | 2015 | Senior | 90 | 7.7 |
| 165 | Reducing mortality in acute kidney injury | J Cardiothorac Vasc Anesth | 2013 | Senior | 90 | 2.3 |
| 166 | Renal blood flow during acute renal failure in man | Blood Purif | 2009 | Senior | 90 | 2.2 |
| 167 | Reducing glycemic variability in intensive care | J Diabetes Sci Technol | 2009 | Senior | 89 | 2.9 |
| 168 | A quantitative analysis of the acidosis of | Crit Care | 2005 | Senior | 88 | 8.8 |
| 169 | The nature and discriminatory value of urinary | Intensive Care Med | 2013 | Senior | 87 | 27.1 |
| 170 | Contrast-enhanced ultrasonography to evaluate | Crit Care | 2014 | Senior | 87 | 8.8 |
| 171 | Severe ischemic early liver injury after cardiac | Ann Thorac Surg | 2002 | Senior | 87 | 3.8 |
| 172 | Conceptual advances and evolving terminology in | Nat Rev Nephrol | 2021 | Senior | 86 | 23.6 |
| 173 | Clinical review: Optimal dose of continuous renal | Crit Care | 2011 | Senior | 86 | 8.8 |
| 174 | Increasing renal blood flow: low-dose dopamine or | Chest | 2004 | Senior | 86 | 10.3 |
| 175 | The influence of volume management on outcome | Curr Opin Crit Care | 2007 | Senior | 86 | 3.5 |
| 176 | A prospective comparative study of moderate | Ren Fail | 1997 | Primary | 86 | 2.9 |
| 177 | Impact of continuous veno-venous hemofiltration | Int J Artif Organs | 2003 | Senior | 85 | 1.4 |
| 178 | A Randomized Trial of Intravenous Amino Acids for | N Engl J Med | 2024 | Senior | 85 | 96.2 |
| 179 | Management of severe acute renal failure in | Nephrol Dial Transplant | 2001 | Senior | 84 | 4.8 |
| 180 | Randomized, double-blind, placebo-controlled | Crit Care Med | 2006 | Senior | 84 | 7.7 |
| 181 | Restrictive fluid management versus usual care in | Intensive Care Med | 2021 | Senior | 84 | 27.1 |
| 182 | Importance of increased ultrafiltration volume | Curr Opin Nephrol Hypertens | 2001 | Senior | 83 | 3 |
| 183 | Randomized Evidence for Reduction of | J Cardiothorac Vasc Anesth | 2017 | Senior | 83 | 2.3 |
| 184 | The effect of albumin concentration on plasma | Anesth Analg | 2007 | Senior | 82 | 5.1 |
| 185 | An assessment of the accuracy of renal blood flow | Intensive Care Med | 2008 | Senior | 82 | 27.1 |
| 186 | Renal replacement therapy in the ICU: | Curr Opin Crit Care | 2018 | Senior | 82 | 3.5 |
| 187 | Changes in blood pressure before the development | Nephrol Dial Transplant | 2009 | Senior | 80 | 4.8 |
| 188 | Paracetamol: a review with specific focus on the | Heart Lung Vessel | 2015 | Senior | 80 | 0.4 |
| 189 | Acute continuous hemodiafiltration: a prospective | Am J Kidney Dis | 1993 | Primary | 79 | 6.8 |
| 190 | A pilot study of high-adsorption hemofiltration | Int J Artif Organs | 2007 | Senior | 78 | 1.4 |
| 191 | Hyperchloremic acidosis in the critically ill: | Anesth Analg | 2006 | Senior | 77 | 5.1 |
| 192 | Changing acute renal failure treatment from | Int J Artif Organs | 1999 | Primary | 77 | 1.4 |
| 193 | Effect of an education programme on the | Intern Med J | 2006 | Senior | 77 | 1.8 |
| 194 | Why is there such a difference in outcome between | Curr Opin Anaesthesiol | 2007 | Primary | 76 | 2.3 |
| 195 | What is a NICE-SUGAR for patients in the | Mayo Clin Proc | 2009 | Primary | 75 | 11 |
| 196 | The effect of low-dose furosemide in critically | J Crit Care | 2017 | Senior | 74 | 2.9 |
| 197 | Treatment of sepsis-associated severe acute renal | Blood Purif | 1995 | Primary | 74 | 2.2 |
| 198 | Prolonged intermittent renal replacement therapy | Crit Care Resusc | 2002 | Primary | 73 | 1.4 |
| 199 | Stress hyperlactatemia modifies the relationship | Crit Care Med | 2014 | Senior | 73 | 7.7 |
| 200 | Glomerular haemodynamics, the renal sympathetic | Nephrol Dial Transplant | 2014 | Senior | 73 | 4.8 |
| 201 | Albumin as a drug: its biological effects beyond | Crit Care Resusc | 2020 | Senior | 72 | 1.4 |
| 202 | Tumor necrosis factor clearances during | ASAIO Trans | 1991 | Primary | 71 | 2.4 |
| 203 | Renal-dose dopamine: from hypothesis to paradigm | J Intensive Care Med | 2005 | Senior | 70 | 2.9 |
| 204 | The impact of post-operative sepsis on mortality | Crit Care | 2017 | Senior | 70 | 8.8 |
| 205 | Consensus development in acute renal failure: The | Curr Opin Crit Care | 2005 | Senior | 70 | 3.5 |
| 206 | Cerebral oxygenation in mechanically ventilated | Resuscitation | 2016 | Senior | 70 | 6.5 |
| 207 | Greater increase in urinary hepcidin predicts | Nephrol Dial Transplant | 2012 | Senior | 70 | 4.8 |
| 208 | Renal replacement therapy intensity for acute | Nephrol Dial Transplant | 2018 | Senior | 68 | 4.8 |
| 209 | Harm of IV High-Dose Vitamin C Therapy in Adult | Crit Care Med | 2020 | Senior | 68 | 7.7 |
| 210 | The Impact of Fluid Balance on the Detection, | J Cardiothorac Vasc Anesth | 2015 | Senior | 68 | 2.3 |
| 211 | Postoperative renal dysfunction after noncardiac | Curr Opin Crit Care | 2017 | Senior | 68 | 3.5 |
| 212 | Clearance of vancomycin during high-volume | Intensive Care Med | 2002 | Senior | 67 | 27.1 |
| 213 | Pleural drainage using central venous catheters | Crit Care | 2003 | Senior | 67 | 8.8 |
| 214 | Pathophysiology of septic acute kidney injury: a | Contrib Nephrol | 2010 | Senior | 67 | 2.3 |
| 215 | The impact of experimental hypoperfusion on | Intensive Care Med | 2010 | Senior | 67 | 27.1 |
| 216 | Calorie intake and patient outcomes in severe | Crit Care | 2014 | Primary | 67 | 8.8 |
| 217 | Postoperative hypothermia and patient outcomes | Anaesthesia | 2011 | Senior | 66 | 6.2 |
| 218 | Paracetamol therapy and outcome of critically ill | Crit Care | 2015 | Senior | 64 | 8.8 |
| 219 | Use of continuous haemodiafiltration: an approach | Am J Nephrol | 1992 | Primary | 64 | 3 |
| 220 | Magnesium status and magnesium therapy in | J Crit Care | 2015 | Senior | 63 | 2.9 |
| 221 | Fluid administration and the kidney | Curr Opin Crit Care | 2013 | Senior | 63 | 3.5 |
| 222 | Fluid administration and the kidney | Curr Opin Crit Care | 2010 | Senior | 63 | 3.5 |
| 223 | Prevention of acute renal failure in the | Nephron Clin Pract | 2003 | Senior | 63 | 1.471 |
| Order | **Short Title** | **Journal** | **Year** | **Role** | **Citations*** | **IF^** |
| 224 | Mortality is Greater in Septic Patients With | Shock | 2017 | Senior | 63 | 3.4 |
| 225 | Renal bioenergetics during early gram-negative | Intensive Care Med | 2012 | Senior | 62 | 27.1 |
| 226 | Correction and Control of Hyperammonemia in Acute | Crit Care Med | 2020 | Senior | 61 | 7.7 |
| 227 | Atelectasis and mechanical ventilation mode | J Crit Care | 2015 | Senior | 61 | 2.9 |
| 228 | The effects of saline or albumin resuscitation on | Crit Care Med | 2006 | Primary | 60 | 7.7 |
| 229 | Online monitoring in continuous renal replacement | Kidney Int Suppl | 1999 | Senior | 60 | 2 |
| 230 | Clinical review: the role of the intensivist and | Crit Care | 2013 | Senior | 59 | 8.8 |
| 231 | Do we know the optimal dose for renal replacement | Kidney Int | 2006 | Primary | 59 | 8.5 |
| 232 | Perioperative fluid prescription, complications | Anaesth Intensive Care | 2010 | Senior | 59 | 1.1 |
| 233 | Cluster randomised crossover trials with binary | Clin Trials | 2015 | Senior | 58 | 2.5 |
| 234 | Establishment of enteral nutrition: prokinetic | Curr Opin Crit Care | 2004 | Senior | 58 | 3.5 |
| 235 | The impact of oxygen and carbon dioxide | Curr Opin Crit Care | 2014 | Senior | 57 | 3.5 |
| 236 | Fluid administration and the kidney | Curr Opin Crit Care | 2013 | Senior | 56 | 3.5 |
| 237 | Fluid administration and the kidney | Curr Opin Crit Care | 2010 | Senior | 56 | 3.5 |
| 238 | Rasburicase therapy in acute hyperuricemia and | Contrib Nephrol | 2005 | Senior | 55 | 2.3 |
| 239 | Circuit lifespan during continuous renal | J Crit Care | 2012 | Senior | 54 | 2.9 |
| 240 | MET: the emergency medical team or the medical | Crit Care Resusc | 2004 | Senior | 54 | 1.4 |
| 241 | Measurement of kidney perfusion in critically ill | Crit Care | 2013 | Senior | 54 | 8.8 |
| 242 | Importance of increased ultrafiltration volume | EDTNA ERCA J | 2002 | Senior | 54 | 1.9 |
| 243 | Towards defining persistent critical illness and | Crit Care Resusc | 2015 | Senior | 54 | 1.4 |
| 244 | Renal replacement therapy in acute renal failure | Best Pract Res Clin Anaesthesiol | 2004 | Senior | 53 | 2.4 |
| 245 | Totem and taboo: fluids in sepsis | Crit Care | 2011 | Senior | 53 | 8.8 |
| 246 | Continuous arteriovenous haemodiafiltration: | Aust N Z J Med | 1990 | Primary | 53 | 0.46 |
| 247 | Effect of continuous venovenous hemofiltration | Crit Care Med | 1994 | Primary | 53 | 7.7 |
| 248 | The SPARK Study: a phase II randomized blinded | Trials | 2010 | Senior | 52 | 2.9 |
| 249 | Severe acute kidney injury not treated with renal | Nephrol Dial Transplant | 2012 | Senior | 52 | 4.8 |
| 250 | Conservative oxygen therapy in mechanically | Resuscitation | 2016 | Senior | 52 | 6.5 |
| 251 | Dialysis in intensive care unit patients with | Clin J Am Soc Nephrol | 2007 | Senior | 51 | 7.2 |
| 252 | Life-threatening sodium valproate overdose: a | Crit Care Med | 2009 | Senior | 51 | 7.7 |
| 253 | Treatment of life-threatening lithium toxicity | Crit Care Med | 1991 | Primary | 51 | 7.7 |
| 254 | Continuous renal replacement in critical illness | Contrib Nephrol | 2007 | Senior | 51 | 2.3 |
| 255 | Increasing the use of an existing medical | Anaesth Intensive Care | 2006 | Senior | 50 | 1.1 |
| 256 | High-volume hemofiltration | Contrib Nephrol | 2001 | Primary | 50 | 2.3 |
| 257 | Septic acute kidney injury: the glomerular | Contrib Nephrol | 2011 | Primary | 50 | 2.3 |
| 258 | A pilot, randomized, double-blind, cross-over | Blood Purif | 2009 | Senior | 50 | 2.2 |
| 259 | Super high flux hemodialysis at high dialysate | Int J Artif Organs | 2004 | Senior | 50 | 1.4 |
| 260 | Techniques of extracorporeal cytokine removal: a | Ren Fail | 2013 | Senior | 49 | 2.9 |
| 261 | Persistent critical illness characterised by | Crit Care Resusc | 2015 | Senior | 48 | 1.4 |
| 262 | qSOFA as predictor of mortality and prolonged ICU | J Crit Care | 2018 | Senior | 48 | 2.9 |
| 263 | Understanding acute kidney injury in sepsis | Intensive Care Med | 2014 | Senior | 48 | 27.1 |
| 264 | Acute kidney injury in patients with influenza A | Intensive Care Med | 2011 | Senior | 48 | 27.1 |
| 265 | Electronic bed weighing vs daily fluid balance | J Crit Care | 2013 | Senior | 48 | 2.9 |
| 266 | Instability of urinary NGAL during long-term | Am J Kidney Dis | 2009 | Senior | 48 | 6.8 |
| 267 | Angiotensin II infusion in COVID-19-associated | Crit Care | 2020 | Senior | 48 | 8.8 |
| 268 | Vasoactive drugs and the kidney | Best Pract Res Clin Anaesthesiol | 2004 | Senior | 48 | 2.4 |
| 269 | Bowel motions in critically ill patients: a pilot | Crit Care Resusc | 2010 | Senior | 47 | 1.4 |
| 270 | Septic acute kidney injury: hemodynamic syndrome, | Crit Care | 2011 | Senior | 47 | 8.8 |
| 271 | Possible strategies to prolong circuit life | Ren Fail | 2002 | Senior | 47 | 2.9 |
| 272 | Sex Differences in Treatment of Adult Intensive | Crit Care Med | 2022 | Senior | 47 | 7.7 |
| 273 | Central venous pressure is a stopping rule, not a | Crit Care Resusc | 2014 | Senior | 47 | 1.4 |
| 274 | Myoglobin clearance during acute continuous | Intensive Care Med | 1991 | Primary | 47 | 27.1 |
| 275 | Persistent critical illness: baseline | Crit Care Resusc | 2019 | Senior | 46 | 1.4 |
| 276 | A comparison of therapeutic hypothermia and | Resuscitation | 2016 | Senior | 46 | 6.5 |
| 277 | Early net ultrafiltration rate and mortality in | Nephrol Dial Transplant | 2021 | Senior | 46 | 4.8 |
| 278 | The epidemiology of bacteriuria and candiduria in | Epidemiol Infect | 2015 | Senior | 46 | 2.4 |
| 279 | Sodium bicarbonate therapy for critically ill | J Crit Care | 2019 | Senior | 46 | 2.9 |
| 280 | Goal-directed resuscitation in septic shock | N Engl J Med | 2015 | Senior | 46 | 96.2 |
| 281 | The acid-base effects of continuous | Int J Artif Organs | 2003 | Senior | 45 | 1.4 |
| 282 | 20% Human Albumin Solution Fluid Bolus | J Cardiothorac Vasc Anesth | 2019 | Senior | 45 | 2.3 |
| 283 | Relative Hypoglycemia in Diabetic Patients With | Crit Care Med | 2020 | Senior | 44 | 7.7 |
| 284 | Micronutrient deficiency in critical illness: an | Intensive Care Med | 2019 | Senior | 44 | 27.1 |
| 285 | Defining the characteristics and expectations of | J Crit Care | 2016 | Senior | 44 | 2.9 |
| 286 | Fluid resuscitation: colloids vs. crystalloids | Blood Purif | 2002 | Primary | 44 | 2.2 |
| 287 | A Double-Blind Randomized Controlled Trial of | Crit Care Med | 2018 | Senior | 44 | 7.7 |
| 288 | Glycated Hemoglobin A1c Levels Are Not Affected | Crit Care Med | 2016 | Senior | 44 | 7.7 |
| 289 | Emergency department rapid response systems: the | Eur J Emerg Med | 2013 | Senior | 43 | 1.8 |
| 290 | Fever in sepsis: is it cool to be hot? | Crit Care | 2014 | Senior | 43 | 8.8 |
| 291 | Amino acid balance with extended daily | Blood Purif | 2012 | Senior | 43 | 2.2 |
| 292 | Introduction of a rapid response system: why we | Crit Care | 2006 | Senior | 42 | 8.8 |
| 293 | Management of early acute renal failure: focus on | Curr Opin Crit Care | 2005 | Primary | 42 | 3.5 |
| 294 | N-Acetylcysteine does not artifactually lower | Nephrol Dial Transplant | 2008 | Senior | 42 | 4.8 |
| 295 | Intravenous amino acid therapy for kidney | J Thorac Cardiovasc Surg | 2019 | Senior | 42 | 5.3 |
| 296 | Coagulation in acutely ill patients with severe | J Crit Care | 2017 | Senior | 42 | 2.9 |
| 297 | Sex differences in illness severity and mortality | J Crit Care | 2021 | Senior | 41 | 2.9 |
| 298 | Are all fluids bad for the kidney? | Curr Opin Crit Care | 2015 | Senior | 41 | 3.5 |
| Order | **Short Title** | **Journal** | **Year** | **Role** | **Citations*** | **IF^** |
| 299 | Current worldwide practice of dialysis dose | Curr Opin Crit Care | 2006 | Senior | 41 | 3.5 |
| 300 | Fluid Bolus Therapy-Based Resuscitation for | Pediatr Crit Care Med | 2015 | Senior | 40 | 3.9 |
| 301 | Pharmacokinetic data support 6-hourly dosing of | Crit Care Resusc | 2019 | Senior | 40 | 1.4 |
| 302 | Preventing cerebral oedema in acute liver | Anaesth Intensive Care | 2014 | Senior | 40 | 1.1 |
| 303 | The kidney in heart failure | Kidney Int Suppl | 1998 | Primary | 40 | 2 |
| 304 | Management of acute renal failure in the | Ren Fail | 1992 | Primary | 40 | 2.9 |
| 305 | Solute mass balance during isovolaemic high | Intensive Care Med | 2003 | Senior | 39 | 27.1 |
| 306 | Epidemiology and Outcomes of Acute Kidney | Am J Nephrol | 2021 | Senior | 39 | 3 |
| 307 | The RENAL (Randomised Evaluation of Normal vs. | Crit Care Resusc | 2009 | Senior | 39 | 1.4 |
| 308 | In vivo catecholamine extraction during | ASAIO Trans | 1991 | Primary | 39 | 2.4 |
| 309 | Basic mechanisms and definitions for continuous | Int J Artif Organs | 1996 | Senior | 38 | 1.4 |
| 310 | Initial and extended use of femoral versus | Am J Kidney Dis | 2014 | Senior | 38 | 6.8 |
| 311 | Urinalysis and pre-renal acute kidney injury: | Crit Care | 2013 | Senior | 38 | 8.8 |
| 312 | Vitamin C measurement in critical illness: | Clin Chem Lab Med | 2020 | Senior | 38 | 3.2 |
| 313 | New CRRT systems: impact on dose delivery | Am J Kidney Dis | 1997 | Senior | 37 | 6.8 |
| 314 | Techniques of extracorporeal cytokine removal: a | Blood Purif | 2012 | Senior | 37 | 2.2 |
| 315 | Acute kidney injury: how can we facilitate | Curr Opin Crit Care | 2011 | Senior | 37 | 3.5 |
| 316 | Daily protein intake and patient outcomes in | Blood Purif | 2014 | Primary | 37 | 2.2 |
| 317 | Idiopathic membranous nephropathy in an | Nephron | 1993 | Primary | 37 | 2.3 |
| 318 | Metabolic and electrolyte disturbance after | Best Pract Res Clin Anaesthesiol | 2015 | Primary | 36 | 2.4 |
| 319 | Clearance of vancomycin during continuous | Crit Care Med | 1990 | Primary | 36 | 7.7 |
| 320 | Associations of fluid amount, type, and balance | Anaesth Intensive Care | 2018 | Senior | 36 | 1.1 |
| 321 | A Pilot, Double-Blind, Randomized, Controlled | J Cardiothorac Vasc Anesth | 2020 | Senior | 36 | 2.3 |
| 322 | Current ventilation practice during general | BMC Anesthesiol | 2014 | Senior | 36 | 2.1 |
| 323 | Extracorporeal blood purification therapy for | Contrib Nephrol | 2001 | Primary | 35 | 2.3 |
| 324 | The epidemiology and outcome of medical emergency | Resuscitation | 2011 | Senior | 35 | 6.5 |
| 325 | Continuous renal replacement therapy and its | Crit Care Resusc | 2020 | Senior | 35 | 1.4 |
| 326 | Potential Impact of the 2016 Consensus | Ann Emerg Med | 2017 | Senior | 35 | 4.5 |
| 327 | Predictors and Outcomes of Cardiac | Heart Lung Circ | 2019 | Senior | 34 | 2.6 |
| 328 | Duration of red blood cells storage and outcome | J Crit Care | 2014 | Senior | 34 | 2.9 |
| 329 | Differential effects of steroids on | Kidney Int | 1984 | Senior | 34 | 8.5 |
| 330 | A feasibility study of functional status and | Anaesth Intensive Care | 2016 | Senior | 34 | 1.1 |
| 331 | Phoxilium vs Hemosol-B0 for continuous renal | J Crit Care | 2013 | Senior | 34 | 2.9 |
| 332 | Retrospective frailty determination in critical | Anaesth Intensive Care | 2019 | Senior | 34 | 1.1 |
| 333 | Contrast-enhanced ultrasound evaluation of the | Ren Fail | 2015 | Senior | 34 | 2.9 |
| 334 | Haemodynamic and biochemical responses to fluid | Crit Care Resusc | 2015 | Senior | 33 | 1.4 |
| 335 | The relationship between hypophosphataemia and | Crit Care Resusc | 2014 | Primary | 33 | 1.4 |
| 336 | Acute kidney injury | Lancet | 2012 | Primary | 33 | 79.3 |
| 337 | Erythropoiesis-stimulating Agents in Critically | Ann Surg | 2017 | Senior | 33 | 10.1 |
| 338 | Open letter to the Executive Director of the | Br J Anaesth | 2014 | Primary | 33 | 6.9 |
| 339 | Open Letter to the Executive Director of the | Acta Anaesthesiol Scand | 2014 | Primary | 33 | 2.9 |
| 340 | Early glycemia and mortality in critically ill | J Crit Care | 2018 | Senior | 32 | 2.9 |
| 341 | A before and after trial of the effect of a | Crit Care Resusc | 2005 | Primary | 32 | 1.4 |
| 342 | Sex Differences in Mortality of ICU Patients | Am J Respir Crit Care Med | 2022 | Senior | 32 | 19.3 |
| 343 | Development and Validation of a Score to Identify | J Cardiothorac Vasc Anesth | 2019 | Senior | 32 | 2.3 |
| 344 | Combined veno-venous bypass and high volume | ASAIO J | 1993 | Primary | 32 | 2.4 |
| 345 | Extracorporeal Ammonia Clearance for | Blood Purif | 2021 | Senior | 32 | 2.2 |
| 346 | Oxygen consumption and lactate release by the | Crit Care Resusc | 2000 | Senior | 32 | 1.4 |
| 347 | Remote ischemic conditioning for kidney | J Crit Care | 2016 | Senior | 32 | 2.9 |
| 348 | A Pilot Study of Angiotensin Ii as Primary | Shock | 2023 | Senior | 32 | 3.4 |
| 349 | Who should manage CRRT in the ICU? The | Am J Kidney Dis | 1997 | Primary | 32 | 6.8 |
| 350 | The systemic and regional hemodynamic effects of | Anesth Analg | 2012 | Senior | 32 | 5.1 |
| 351 | The obesity paradox and hypoglycemia in | Crit Care | 2021 | Senior | 31 | 8.8 |
| 352 | A technique for the simultaneous measurement of | Crit Care Resusc | 2007 | Senior | 31 | 1.4 |
| 353 | Acute continuous hemofiltration with dialysis: | Crit Care Med | 1992 | Primary | 31 | 7.7 |
| 354 | Cardiac surgery-associated acute kidney injury | Int J Artif Organs | 2008 | Senior | 31 | 1.4 |
| 355 | A pilot study of pulse contour cardiac output | Crit Care Resusc | 2005 | Senior | 31 | 1.4 |
| 356 | Techniques of extracorporeal cytokine removal: a | Int J Artif Organs | 2013 | Senior | 31 | 1.4 |
| 357 | Fluid resuscitation and the septic kidney | Curr Opin Crit Care | 2006 | Senior | 31 | 3.5 |
| 358 | Femoral Access and Delivery of Continuous Renal | Blood Purif | 2016 | Primary | 31 | 2.2 |
| 359 | Gender differences in mortality and quality of | J Crit Care | 2020 | Senior | 30 | 2.9 |
| 360 | The effect of PVC packaging on the acidity of | Anaesth Intensive Care | 2000 | Senior | 30 | 1.1 |
| 361 | A pilot assessment of alpha-stat vs pH-stat | J Crit Care | 2015 | Senior | 30 | 2.9 |
| 362 | Patient characteristics, ICU-specific supports, | J Crit Care | 2019 | Senior | 29 | 2.9 |
| 363 | A Phase II Cluster-Crossover Randomized Trial of | Am J Respir Crit Care Med | 2021 | Senior | 29 | 19.3 |
| 364 | Subacute kidney injury in hospitalized patients | Clin J Am Soc Nephrol | 2014 | Senior | 29 | 7.2 |
| 365 | Vitamin C, Hydrocortisone and Thiamine in | Crit Care Resusc | 2019 | Senior | 29 | 1.4 |
| 366 | A survey of ward nurses attitudes to the | Aust Crit Care | 2012 | Senior | 29 | 2.2 |
| 367 | Noradrenaline: friend or foe? | Heart Lung Circ | 2003 | Primary | 29 | 2.6 |
| 368 | Effect of pump prime on acidosis, | Anaesth Intensive Care | 2009 | Senior | 28 | 1.1 |
| 369 | The Australian approach to peri-operative fluid | Curr Opin Anaesthesiol | 2012 | Senior | 28 | 2.3 |
| 370 | Are systematic reviews and meta-analyses still | Intensive Care Med | 2018 | Senior | 28 | 27.1 |
| 371 | Coupled plasma filtration adsorption | Intensive Care Med | 2003 | Primary | 28 | 27.1 |
| 372 | Premature circuit clotting due to likely | Blood Purif | 2010 | Senior | 28 | 2.2 |
| 373 | Outcome comparisons of intermittent and | Int J Artif Organs | 2008 | Senior | 28 | 1.4 |
| Order | **Short Title** | **Journal** | **Year** | **Role** | **Citations*** | **IF^** |
| 374 | Coupled plasma filtration adsorption | Blood Purif | 2002 | Primary | 28 | 2.2 |
| 375 | Bubble chamber clotting during continuous renal | Blood Purif | 2012 | Senior | 28 | 2.2 |
| 376 | Urine abnormalities in acute kidney injury and | Contrib Nephrol | 2010 | Senior | 28 | 2.3 |
| 377 | The case of rapid response systems: are | Crit Care Med | 2007 | Senior | 28 | 7.7 |
| 378 | Loop diuretic therapy in the critically ill: a | Crit Care Resusc | 2015 | Senior | 27 | 1.4 |
| 379 | Normothermic extracorporeal perfusion of isolated | Crit Care Resusc | 2012 | Primary | 27 | 1.4 |
| 380 | Does fluid management affect the occurrence of | Curr Opin Anaesthesiol | 2017 | Senior | 26 | 2.3 |
| 381 | Epidemiology of early Rapid Response Team | Australas Emerg Nurs J | 2016 | Senior | 26 | 2.1 |
| 382 | Why we must cluster and cross over | Crit Care Resusc | 2013 | Primary | 26 | 1.4 |
| 383 | Intravenous fluid administration and monitoring | Nurs Health Sci | 2012 | Senior | 25 | 1.5 |
| 384 | Borderline anaemia and postoperative outcome in | Anaesthesia | 2020 | Senior | 25 | 6.2 |
| 385 | Changes in intravenous fluid use patterns in | Crit Care Resusc | 2016 | Senior | 25 | 1.4 |
| 386 | Patterns and Mechanisms of Artificial Kidney | Blood Purif | 2016 | Senior | 25 | 2.2 |
| 387 | Near-infrared spectroscopy of the thenar | Crit Care Resusc | 2012 | Senior | 24 | 1.4 |
| 388 | Adequacy of dialysis in the acute renal failure | Int J Artif Organs | 1996 | Primary | 24 | 1.4 |
| 389 | Mortality in patients with hypovolemic shock | JAMA | 2014 | Primary | 24 | 63.1 |
| 390 | Renal replacement therapy in the intensive care | Crit Care Resusc | 1999 | Primary | 24 | 1.4 |
| 391 | Characteristics, incidence and outcome of | Respirology | 2017 | Senior | 24 | 5 |
| 392 | Variability in Serum Sodium Concentration and | Neurocrit Care | 2021 | Senior | 24 | 4.2 |
| 393 | Decreased mean perfusion pressure as an | Heart Vessels | 2020 | Senior | 24 | 2.4 |
| 394 | Biochemical effects of phosphate-containing | Blood Purif | 2012 | Senior | 24 | 2.2 |
| 395 | Comparison of three methods to estimate plasma | Anaesth Intensive Care | 2001 | Senior | 24 | 1.1 |
| 396 | Risk factors for major adverse kidney events in | Clin Kidney J | 2021 | Senior | 23 | 3.2 |
| 397 | Mediators of the Impact of Hourly Net | Crit Care Med | 2020 | Senior | 23 | 7.7 |
| 398 | Circuit start during continuous renal replacement | Blood Purif | 2011 | Senior | 23 | 2.2 |
| 399 | Simple translational equations to compare illness | J Crit Care | 2013 | Senior | 23 | 2.9 |
| 400 | Rapid response teams improve outcomes: we are not | Intensive Care Med | 2016 | Senior | 23 | 27.1 |
| 401 | Why do multicenter randomized controlled trials | Minerva Anestesiol | 2019 | Senior | 23 | 1.7 |
| 402 | 30-Day Outcomes Post Veno-Arterial Extra | Heart Lung Circ | 2020 | Senior | 23 | 2.6 |
| 403 | Prevention of renal dysfunction in postoperative | Curr Opin Crit Care | 2014 | Senior | 23 | 3.5 |
| 404 | Accuracy of non-invasive body temperature | Crit Care Resusc | 2021 | Senior | 23 | 1.4 |
| 405 | Acute kidney disease and the community | Lancet | 2016 | Senior | 23 | 79.3 |
| 406 | Point-of-care measurement of serum creatinine in | Ren Fail | 2012 | Senior | 22 | 2.9 |
| 407 | A prospective study of continuous venovenous | J Intensive Care Med | 1995 | Primary | 22 | 2.9 |
| 408 | Overview of the study protocols and statistical | Crit Care Resusc | 2015 | Senior | 22 | 1.4 |
| 409 | Ammonia Clearance with Different Continuous Renal | Blood Purif | 2022 | Senior | 22 | 2.2 |
| 410 | The haemodynamic effects of bolus versus slower | J Crit Care | 2017 | Senior | 22 | 2.9 |
| 411 | Effect of Furosemide on Urinary Oxygenation in | Blood Purif | 2019 | Senior | 22 | 2.2 |
| 412 | Predicting Acute Kidney Injury After Cardiac | J Cardiothorac Vasc Anesth | 2021 | Senior | 22 | 2.3 |
| 413 | Perioperative renal protection | Curr Opin Crit Care | 2018 | Senior | 22 | 3.5 |
| 414 | Continuous renal replacement techniques | Contrib Nephrol | 2001 | Senior | 22 | 2.3 |
| 415 | Insertion side, body position and circuit life | Blood Purif | 2011 | Senior | 22 | 2.2 |
| 416 | Extended normothermic extracorporeal perfusion of | Crit Care Resusc | 2014 | Primary | 21 | 1.4 |
| 417 | In-hospital cardiac arrest epidemiology in a | Br J Hosp Med (Lond) | 2017 | Senior | 21 | 0.8 |
| 418 | Circuit Survival during Continuous Venovenous | Blood Purif | 2020 | Senior | 21 | 2.2 |
| 419 | ICU-Based Renal Replacement Therapy | Crit Care Med | 2021 | Primary | 21 | 7.7 |
| 420 | Impact of unit-wide chlorhexidine bathing in | Crit Care Resusc | 2018 | Senior | 21 | 1.4 |
| 421 | Hemoadsorption: consensus report of the 30th | Nephrol Dial Transplant | 2024 | Primary | 21 | 4.8 |
| 422 | Urinary hepcidin: an inverse biomarker of acute | Curr Opin Crit Care | 2010 | Senior | 21 | 3.5 |
| 423 | Paracetamol therapy for septic critically ill | Crit Care Resusc | 2011 | Senior | 21 | 1.4 |
| 424 | Cost-Effectiveness of Erythropoietin in Traumatic | J Neurotrauma | 2019 | Senior | 21 | 4.2 |
| 425 | Early Osmotherapy in Severe Traumatic Brain | J Neurotrauma | 2020 | Senior | 21 | 4.2 |
| 426 | Differential clinical characteristics, management | Intern Med J | 2019 | Senior | 21 | 1.8 |
| 427 | Pharmacokinetics of Magnesium Bolus Therapy in | J Cardiothorac Vasc Anesth | 2018 | Senior | 21 | 2.3 |
| 428 | A rapid intravenous phosphate replacement | Crit Care Resusc | 2004 | Senior | 21 | 1.4 |
| 429 | Haemoglobin concentration and volume of | Crit Care | 2018 | Senior | 20 | 8.8 |
| 430 | Contrast-enhanced ultrasound evaluation of renal | Intensive Care Med Exp | 2014 | Senior | 20 | 2.1 |
| 431 | Cine phase-contrast magnetic resonance imaging | Contrib Nephrol | 2010 | Senior | 20 | 2.3 |
| 432 | Normotensive ischemic acute renal failure | N Engl J Med | 2007 | Primary | 20 | 96.2 |
| 433 | Perioperative Statins in Cardiac Surgery and | JAMA | 2016 | Primary | 20 | 63.1 |
| 434 | Fluid resuscitation and the septic kidney: the | Contrib Nephrol | 2007 | Senior | 20 | 2.3 |
| 435 | Characteristics, incidence and outcome of | J Crit Care | 2018 | Senior | 20 | 2.9 |
| 436 | Automated electronic monitoring of circuit | Crit Care Resusc | 2015 | Senior | 20 | 1.4 |
| 437 | A technique for the measurement of renal ATP in a | Int J Artif Organs | 2005 | Senior | 19 | 1.4 |
| 438 | Coagulation abnormalities, bleeding, thrombosis, | J Gastroenterol Hepatol | 2020 | Senior | 19 | 4.2 |
| 439 | Haemodynamic Impact of a slower pump speed at | Blood Purif | 2012 | Senior | 19 | 2.2 |
| 440 | An assessment of the triage performance of the | Resuscitation | 2013 | Senior | 19 | 6.5 |
| 441 | How to feed patients with renal dysfunction | Curr Opin Crit Care | 2000 | Primary | 19 | 3.5 |
| 442 | Glycaemic control in Australia and New Zealand | Crit Care | 2013 | Senior | 19 | 8.8 |
| 443 | Continuous bladder urinary oxygen tension as a | Crit Care | 2022 | Senior | 19 | 8.8 |
| 444 | The prognostic value of the strong ion gap in | J Crit Care | 2016 | Senior | 19 | 2.9 |
| 445 | Low-Concentration Norepinephrine Infusion for | Anesth Analg | 2022 | Senior | 19 | 5.1 |
| 446 | Efficacy and Safety of Parenteral High-Dose | Pediatr Crit Care Med | 2021 | Senior | 19 | 3.9 |
| 447 | Early metabolic acidosis in critically ill | Crit Care Resusc | 2021 | Senior | 19 | 1.4 |
| 448 | Comparison of Thromboelastography and | Clin Appl Thromb Hemost | 2020 | Senior | 19 | 2 |
| Order | **Short Title** | **Journal** | **Year** | **Role** | **Citations*** | **IF^** |
| 449 | How to feed patients with renal dysfunction | Blood Purif | 2002 | Primary | 19 | 2.2 |
| 450 | Novel renal biomarkers of acute kidney injury and | Intern Med J | 2021 | Primary | 19 | 1.8 |
| 451 | How I prescribe continuous renal replacement | Crit Care | 2021 | Senior | 19 | 8.8 |
| 452 | Perioperative renal failure in elderly patients | Curr Opin Anaesthesiol | 2015 | Senior | 19 | 2.3 |
| 453 | Coupled plasma filtration adsorption: rationale, | Blood Purif | 2003 | Senior | 18 | 2.2 |
| 454 | Understanding the rationale for parenteral | Crit Care Resusc | 2018 | Senior | 18 | 1.4 |
| 455 | Effects of saline or albumin resuscitation on | Crit Care Resusc | 2009 | Primary | 18 | 1.4 |
| 456 | Combined acute respiratory and renal failure: | Resuscitation | 1994 | Primary | 18 | 6.5 |
| 457 | Coupled plasma filtration adsorption: rationale, | Contrib Nephrol | 2004 | Senior | 18 | 2.3 |
| 458 | Circulation of the continuous artificial kidney: | Blood Purif | 1997 | Primary | 18 | 2.2 |
| 459 | Hemofiltration in sepsis: where do we go from | Crit Care | 2000 | Senior | 18 | 8.8 |
| 460 | Patient characteristics, incidence, technique, | J Crit Care | 2018 | Senior | 17 | 2.9 |
| 461 | Sex Differences in Vital Organ Support Provided | Crit Care Med | 2024 | Senior | 17 | 7.7 |
| 462 | Relationship between blood flow, access catheter | Contrib Nephrol | 2004 | Senior | 17 | 2.3 |
| 463 | Acute renal failure and sepsis | N Engl J Med | 2004 | Primary | 17 | 96.2 |
| 464 | Interventions affecting mortality in critically | J Crit Care | 2017 | Senior | 17 | 2.9 |
| 465 | The systemic inflammatory response syndrome | J Crit Care | 2018 | Senior | 17 | 2.9 |
| 466 | Continuous Renal Replacement Therapy: The | Am J Respir Crit Care Med | 2021 | Senior | 17 | 19.3 |
| 467 | Characteristics and outcomes of patients with | Intern Med J | 2019 | Senior | 17 | 1.8 |
| 468 | Amino Acid Infusion for Perioperative Functional | J Cardiothorac Vasc Anesth | 2024 | Senior | 17 | 2.3 |
| 469 | Twenty percent human albumin solution fluid bolus | Intensive Care Med | 2024 | Senior | 16 | 27.1 |
| 470 | Hemodynamic management of septic shock | Minerva Anestesiol | 2015 | Senior | 16 | 1.7 |
| 471 | Pharmacodynamics of intravenous frusemide bolus | Crit Care Resusc | 2017 | Senior | 16 | 1.4 |
| 472 | Point-of-care testing during medical emergency | Resuscitation | 2012 | Senior | 16 | 6.5 |
| 473 | Coronary Artery Bypass Surgery Without Saphenous | J Am Coll Cardiol | 2022 | Senior | 16 | 21.7 |
| 474 | Novel biomarkers of acute kidney injury: ready | Curr Opin Crit Care | 2010 | Senior | 16 | 3.5 |
| 475 | Characteristics, incidence, and outcome of | J Crit Care | 2018 | Senior | 16 | 2.9 |
| 476 | Defining the vital condition for organ donation | Philos Ethics Humanit Med | 2007 | Primary | 16 | 1.9 |
| 477 | Differential effects of isotonic and hypotonic 4% | Crit Care Resusc | 2018 | Senior | 16 | 1.4 |
| 478 | Intensity of early correction of hyperglycaemia | Crit Care Resusc | 2017 | Senior | 16 | 1.4 |
| 479 | Sodium bicarbonate infusion in patients | Clin Transplant | 2016 | Senior | 16 | 1.9 |
| 480 | Quo vadis CRRT? | Kidney Int Suppl | 1998 | Senior | 15 | 2 |
| 481 | Normothermic extracorporeal human liver perfusion | Crit Care Resusc | 2013 | Primary | 15 | 1.4 |
| 482 | Recent trials in critical care nephrology | Contrib Nephrol | 2010 | Primary | 15 | 2.3 |
| 483 | Hidden evidence to the West: multicentre, | Intensive Care Med | 2004 | Primary | 15 | 27.1 |
| 484 | Renal outcomes according to renal replacement | Crit Care | 2022 | Senior | 15 | 8.8 |
| 485 | ACE inhibitors and angiotensin receptor blockers | Crit Care | 2024 | Senior | 15 | 8.8 |
| 486 | Primary fluid bolus therapy for | Crit Care Resusc | 2015 | Senior | 15 | 1.4 |
| 487 | The outcome of critically ill elderly patients | Int J Artif Organs | 1994 | Primary | 15 | 1.4 |
| 488 | Cause and Timing of Death and Subgroup | J Neurotrauma | 2018 | Senior | 15 | 4.2 |
| 489 | Neutrophil gelatinase-associated lipocalin after | Biomarkers | 2014 | Senior | 15 | 2.6 |
| 490 | Fluid balance error in continuous renal | Int J Artif Organs | 2007 | Senior | 15 | 1.4 |
| 491 | An Exploratory Analysis of the Association | Ann Am Thorac Soc | 2022 | Senior | 15 | 6.3 |
| 492 | Rapid response team review of hemodynamically | J Crit Care | 2019 | Senior | 15 | 2.9 |
| 493 | Hypoglycemia in sepsis: biomarker, mediator, or | Crit Care Med | 2011 | Primary | 15 | 7.7 |
| 494 | Intensive care implications of epidemic | Crit Care Resusc | 2018 | Senior | 14 | 1.4 |
| 495 | Glycocalyx damage biomarkers in healthy controls, | Biomarkers | 2020 | Senior | 14 | 2.6 |
| 496 | Oxygen administration and monitoring for ward | Intern Med J | 2011 | Senior | 14 | 1.8 |
| 497 | Sodium glucose co-transporter-2 inhibitors in | Crit Care | 2023 | Senior | 14 | 8.8 |
| 498 | Platelet Transfusion After Cardiac Surgery | J Cardiothorac Vasc Anesth | 2023 | Senior | 14 | 2.3 |
| 499 | Nasal high-flow oxygen therapy in ICU: A | Aust Crit Care | 2016 | Senior | 14 | 2.2 |
| 500 | Defining ICD-10 surrogate variables to estimate | BMC Geriatr | 2022 | Senior | 14 | 2.7 |

* Google Scholar citation at July 1 2025

^ IF = Journal Impact factor in 2024
